# Early Pubertal Development Is a Risk Factor for Psychotic-Like Experiences in Boys and Girls

**DOI:** 10.1016/j.bpsgos.2025.100647

**Published:** 2025-10-31

**Authors:** Eric R. Larson, Natasha Chaku, Alexandra Moussa-Tooks

**Affiliations:** a Department of Psychological and Brain Sciences, Indiana University Bloomington, Bloomington, Indiana; b Program in Neuroscience, Indiana University Bloomington, Bloomington, Indiana; c Department of Psychiatry, Indiana University School of Medicine, Indianapolis, Indiana

**Keywords:** ABCD Study, Adrenarche, Gonadarche, Psychotic-like experiences, Puberty, Timing

## Abstract

**Background:**

Puberty has long been identified as a risk factor for psychosis, although retrospective, cross-sectional, and single-sex indicators of puberty have limited our ability to pinpoint biopsychosocial mechanisms contributing to risk. The current study determined whether individual differences in the timing (onset) and tempo (pace) of pubertal development conferred risk for psychotic-like experiences (PLEs) in youth across biological sex.

**Methods:**

Data included 11,758 youths (6134 boys and 5624 girls) from the ABCD (Adolescent Brain Cognitive Development) Study (average age = 9.9 years at baseline, 12.9 years at 3-year follow-up). Pubertal timing and tempo (overall, adrenarche, gonadarche) were derived from sex-specific linear mixed-effects models using the Pubertal Development Scale. Sex-specific negative binomial multilevel models estimated effects of categorical and continuously measured pubertal timing and tempo and their interaction on year-3 PLEs per the Prodromal Questionnaire-Brief Child.

**Results:**

In both sexes, earlier pubertal timing was associated with elevated PLEs (βs = 0.23 to 0.31), and later pubertal timing was associated with fewer PLEs (βs = −0.22 to −0.52) relative to on-time peers. In boys only, faster pubertal tempo was associated with fewer PLEs relative to on-track peers (βs = −0.21 to −0.30). Analyses with continuous pubertal timing and tempo demonstrated an association between earlier adrenarchal timing and more PLEs in girls only (β = −0.21) and an interaction between adrenarchal timing and tempo in boys only (β = −0.80).

**Conclusions:**

Early pubertal timing in both sexes and faster pubertal tempo in males increases PLEs. Understanding the unique experiences associated with a youth’s pubertal maturation, particularly adrenarche, can advance identification and prevention efforts for children and adolescents at greatest clinical risk.

## Plain Language Summary

Aberrant puberty is a putative risk factor for psychosis. Retrospective, cross-sectional, and single-sex indicators of puberty oversimplify this multiyear biopsychosocial process. In 11,718 youths from the ABCD (Adolescent Brain Cognitive Development) Study, we derived measures of pubertal timing and tempo. Using sex-specific negative binomial multilevel models, we demonstrated that early pubertal timing in boys and girls and fast tempo in boys only are associated with increased endorsement of psychotic-like experiences 3 years later. Findings suggest a central role of social and hormonal factors in the emergence of psychotic-like experiences, which can advance identification and prevention efforts for youth at greatest risk.

Psychotic disorders such as schizophrenia are distressing and impairing, affecting an estimated 3% of the population and imposing significant social, wellness, and financial burden on individuals, families, and their communities (1). Psychotic symptoms become notable in late adolescence and early adulthood, with some sex differences (e.g., in men, an earlier age of onset, more subclinical symptoms, and longer, more severe, and recurrent episodes) (1). These patterns suggest that developmental processes shape some of the risk for psychosis. Indeed, psychotic disorders are neurodevelopmental in nature. There has been an uptick in the investigation of early manifestations of the disorder including psychotic-like experiences (PLEs) (2, 3, 4). PLEs capture subclinical manifestations of hallucinations, delusions, or thought disturbances (5). PLEs are experienced by approximately 20% to 60% of youth (6), with meta-analytic evidence suggesting a 4-fold increase in risk for developing psychosis spectrum disorders, with lower estimates for internalizing and externalizing disorders (7,8). The experience of PLEs, especially when they persist and are distressing, is linked to later psychopathology, highlighting the clinical meaningfulness of these phenomena (5).

Importantly, and consistent with the neurodevelopmental hypothesis of schizophrenia (2, 3, 4), PLEs begin to emerge throughout middle childhood and adolescence, coinciding with a critical period of development, i.e., puberty or the process of reaching reproductive maturity (9). Puberty comprises two interrelated processes. Gonadarche (i.e., the re-activation of the hypothalamic-pituitary-gonadal axis after a period of relative quiescence in childhood) marks the increased production of gonadal sex steroid hormones, primarily estradiol in girls and testosterone in boys. Adrenarche (i.e., the maturation of the adrenal glands and the hypothalamic-pituitary-adrenal [HPA] axis) marks the rise in adrenal androgens such as dehydroepiandrosterone (DHEA) in both sexes (10). Adrenarche typically precedes gonadarche and is associated with early physical changes such as body odor, acne, and pubic hair, while gonadarche is responsible for the development of primary and secondary sex characteristics (11).

Increases in sex steroid hormones during puberty orchestrate rapid and nonlinear changes in body shape and size. These physical changes are often accompanied by experiences of bodily unfamiliarity that mirror anomalous bodily experiences commonly reported in psychosis (12). Sex hormones also cross the blood-brain barrier and exert activational (i.e., short-term) and organizational (i.e., long-term) effects on the HPA axis and neural circuitry in the limbic system and prefrontal cortex (13). The HPA axis and these brain regions are implicated in stress reactivity, emotion regulation, reward sensitivity, and cognitive control, processes that are well established as aberrant in psychotic disorders (1,14). In fact, individuals with psychosis and at clinical high risk for psychosis have been reported to exhibit high levels of cortisol compared with nonpsychiatric control groups, suggesting that pubertal changes may be an important mechanism underlying this phenomenon (15, 16, 17). Physical and cognitive changes also drive psychosocial maturation as youth refine their ways of thinking about themselves and adapt to new social expectations from peers, parents, and society. These evolving social contexts may exacerbate feelings of alienation, self-consciousness, or interpersonal stress, all of which are psychosocial correlates of PLEs (15, 16, 17).

Given the critical biological, cognitive, and social roles that puberty plays over the lifespan and in clinical outcomes, it is unsurprising that pubertal development has long been investigated in the context of psychotic disorders as a means of understanding sex differences in symptomology, disorder onset, and prognosis (18,19). Early work linked the onset of the first psychotic episode in women to their first menses (20). This same group then suggested that pubertal development was more strongly assosiated with positive symptomology, especially in males (21). Recently, more sophisticated studies have found that earlier menarche (i.e., age at first menses) is associated with later onset of schizophrenia in women (22,23). This work directly called for investigation of processes related to gonadarche and adrenarche. Accordingly, individuals with psychosis exhibit fewer positive symptoms with higher estrogen levels and fewer negative symptoms with higher amounts of testosterone (14). However, findings are mixed, likely due to the cross-sectional, retrospective, and female-centric nature of the extant literature. For example, a recent study found that earlier menarche is associated with higher severity of PLEs, and other studies have documented null or opposing effects (24, 25, 26, 27).

Conflicting evidence in the extant literature suggests that it is imperative to investigate more complex features of puberty. For example, pubertal timing (i.e., onset compared with same-age, same-sex peers) and pubertal tempo (i.e., how quickly or slowly youth progress through puberty) index individual differences in when and how quickly youth mature, respectively (9,28). Timing and tempo are estimated using multiple longitudinal assessments of pubertal status (i.e., gonadarchal: menarche and breast development in girls, genital development and voice changes in boys; adrenarchal: skin changes and hair growth in both) and reflect a complex cascade of pubertal milestones and hormonal processes that occur in both sexes (29). Pubertal timing and tempo also capture aspects of the social environment (i.e., how youth appear relative to others), offering a window into how biological maturation interacts with the social context to shape risk for PLEs. Thus, timing and tempo can be used to understand which aspects of puberty (e.g., the age of hormonal increase, the duration and intensity of sex steroid exposure, or adrenal vs. gonadal processes) are most relevant for PLEs.

Earlier timing in particular has been associated with the onset of subsequent mental health problems via biological, cognitive, and psychosocial mechanisms. The hormonal hypothesis suggests that earlier timing is linked to worse mental health outcomes because early developers experience prolonged exposure to sex hormones over the lifespan (15). The maturational disparity hypothesis posits that early developers experience a developmental mismatch between their rapidly developing physical body and generally immature cognitive and psychosocial capacities, which leads to worse mental health outcomes (30). Additional theoretical perspectives suggest that any off-time development (e.g., developing earlier or later than one’s peers) is associated with worse psychosocial and mental health outcomes due to heightened social comparisons (31). While a robust literature has linked earlier timing and faster tempo to increased risk for a wide range of physical and mental health disorders in both sexes (32,33), associations with PLEs are currently unknown.

Using data from the ABCD (Adolescent Brain Cognitive Development) Study, we examined the main effects of pubertal timing and tempo and their interactions on PLEs assessed 3 years later. We assessed overall pubertal timing and tempo and then independently investigated associations with adrenarche and gonadarche to clarify the relative contributions of different hormonal axes. Pubertal timing and tempo were considered as categorical variables (representing “early,” “on-time,” and “late” social categories) and continuous variables (representing absolute age of onset and pace of progression) to clarify whether associations between timing and PLEs were due to maturational deviance, hormonal influences, or off-time development. We hypothesized that earlier timing and faster tempo would be associated with higher reported PLEs in adolescence and that together, earlier timing and faster tempo would predict the most risk. Additionally, we expected that these associations would be driven by gonadal effects and would be consistent across both categorical and continuous measures of pubertal timing.

## Methods and Materials

The ABCD Study is a longitudinal population-based cohort study of 11,875 youth distributed among 21 research sites across the United States. Data collected through the ABCD Study have been approved by an institutional review board (IRB), as described in Auchter *et al.* (34,35). Briefly, many ABCD Study data collection sites cede approval to a central IRB hosted at the University of California San Diego, while others obtain local IRB approval. All caregivers of participants provided written informed consent, and all youth provided assent to participate.

### Sample

Data from 11,758 (6134 boys, 5624 girls) participants in the ABCD Study were used in the current analyses (Data Release 5.1, https://doi.org/10.15154/z563-zd24). Inclusion and exclusion criteria per the ABCD guidelines have been described extensively (5,34,36). Notable exclusion criteria for the current study included not fluent in English; a history of major neurological disorders, traumatic brain injury, or extreme prematurity (gestational age <28 weeks, birth weight <1200 g); and a current formal diagnosis of schizophrenia, moderate to severe autism spectrum disorder, intellectual disability, or substance use disorder. We used data from baseline (ages 9–10) to the 3-year follow-up (ages 12–13 years) in the current study.

### Pubertal Development

Pubertal development was indexed yearly via the Pubertal Development Scale (PDS) (37). The PDS is composed of 3 sex-neutral items and 2 sex-specific items, each assessing a different pubertal milestone (e.g., breast development for girls, vocal changes for boys, changes in body hair growth for both). Items were rated on a 4-point scale (1 = has not yet begun, 2 = has barely started, 3 = is definitely underway, 4 = seems complete) except for menarche (e.g., first menses), which was rated as 1 = has not yet started or 4 = seems complete. Items were averaged to create a composite score of pubertal development at each wave by sex when youth completed at least 4 of 5 items (38). Then adrenal and gonadal subscale scores were created at each wave by averaging across the adrenal (2 items: body hair, skin changes) and gonadal (2 items per sex, girls: breast development, menarche; boys: voice changes, facial hair growth) items, respectively. Youth had to complete both items for adrenarche to be included in adrenal analyses and both items for gonadarche to be included in the gonadal analyses. Given that youth are better reporters of their own development during mid- and late adolescence (9), analyses were conducted with youth-reported PDS.

### Psychotic-Like Experiences

Youth self-reported PLEs were assayed with the Prodromal Questionnaire-Brief Child Version (PQ-BC) (39). This 21-item questionnaire indexes positive psychotic experiences indicating the presence (0 = absent, 1 = present) and associated distress (0 = not endorsed, 1 = endorsed with no distress, 2 − 6 = 1 + distress score) of these experiences and has been validated for use in children in the ABCD sample (39). The current study used the sum score (range = 0–21) and distress score (range = 0–126) of the PQ-BC at the 3-year follow-up.

### Covariates

We included covariates known to influence pubertal timing and tempo and/or PLEs. Premature birth (40) was extracted from the Developmental History Questionnaire (41, 42, 43), reflecting the number of weeks premature the child was born (1–12, >12). Caregiver education from the Demographics Survey was used as an index of socioeconomic status (29,44). Family history of psychosis was assessed with the Family History Assessment (45,46) and scored as the number of family members who reported a history of seeing visions, hearing voices, or thinking that people were spying on or plotting against them. For girls, use of a hormonal contraceptive at any study wave (reported on the PDS) was included (0 = no use, 1 = use). Baseline PLE sum and distress scores were included as covariates in models evaluating sum and distress effects, respectively.

### Analytic Plan

Following previous research (28,47), only adolescents with at least 2 assessments of pubertal development were included in subsequent analyses. This removed 1.2% of all girls and 2.1% of all boys. We derived estimates of timing and tempo from linear mixed-effect models separately by sex using the average pubertal development score at each wave in SAS (48). This linear mixed effects model for overall PDS can be represented as:

(1) $$\text{PDS}=b_{0i}+b_{1i}\times {\text{age}}_{\text{it}}+r_{it}\text{,}$$

where b_0*i*_ represents the intercept, b_1*i*_ represents the slope, and r_*it*_ is the residual for an individual *i* at assessment *t*. Individual estimates of timing and tempo were calculated from this model using Bayes empirical estimates (47,49). Specifically, timing (i.e., the age at which puberty began) was conceptualized as age at PDS 2.0, and tempo was conceptualized as change in PDS over time (i.e., the rate at which individuals progressed through PDS stages). Thus, youth who entered PDS = 2.0 at age 10 would be considered to have earlier timing than youth who entered PDS = 2.0 at age 13; similarly, youth who experienced more stages of growth in the same number of years would be considered to have a faster tempo than those who experienced fewer stages of growth during the same period. Timing and tempo were derived using the overall PDS and then separately for each hormonal axis (adrenal vs. gonadal).

Subsequent statistical analyses were conducted in R version 4.2.0. First, we assessed differences between boys and girls in pubertal development, PLEs, and covariates via *t* tests and χ^2^ tests of equivalence. Then, we estimated a series of negative binomial multilevel models [*glmmTMB* package (50)] to assess the effect of pubertal timing, pubertal tempo, and their interaction on year-3 PLEs. We used negative binomial models because 1) scores on the PQ-BC are positively skewed toward the floor of zero, and 2) these models generally fit the data better than zero-inflated negative binomial and Poisson models.

In the first set of analyses, we estimated associations between categorical pubertal timing (i.e., <1 SD = “early timing,” ±1 SD = “on-time,” >1 SD = “late timing”) and tempo (i.e., <1 SD = “slower pace,” ±1 SD = “on-track pace,” >1 SD = “faster pace”) and year-3 PLEs using normative timing and tempo (±1 SD) as the reference group. Analyses were conducted using an overall measure of categorical pubertal timing and tempo and then separately by hormonal axis (adrenal vs. gonadal). In the second set of analyses, we estimated associations between a continuous metric of pubertal timing and tempo, as well as their interaction, and year-3 PLEs. Analyses were again conducted using an overall measure of continuous pubertal timing and tempo and then separately by hormonal axis (adrenal vs. gonadal).

All analyses were conducted separately in boys and girls given normative differences in pubertal development (9); study site and family membership were included as nested random effects in all models.

## Results

**Figure 1:**
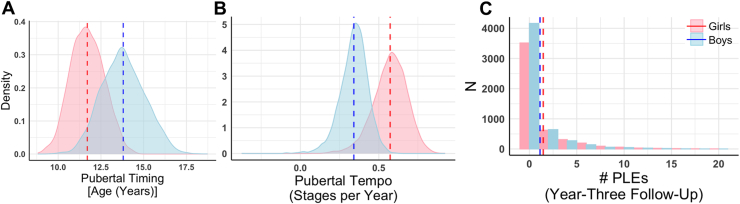
**(A)** Girls reported earlier overall pubertal timing than boys. **(B)** Boys reported slower pubertal tempo than girls. **(C)** Psychotic-like experiences (PLEs) (number of items endorsed on the Prodromal Questionnaire-Brief Child Version) at year 3 were non-normally distributed in the sample, with girls endorsing slightly more PLEs than boys (1.12 vs. 1.16). Dotted lines indicate mean scores for each sex. While not displayed here, distributions of timing and tempo by axis (e.g., adrenarche and gonadarche) evidence similar patterns (i.e., earlier timing in girls, slower tempo in boys).

The sample was majority White, and most caregivers were at least high school educated (Table 1). There were no meaningful demographic differences between boys and girls. Although age was significantly different between boys and girls, this equated to an approximately 1-month difference at baseline and follow-up (Cohen’s *d*: baseline = 0.04, year 3 = 0.06). As expected, pubertal timing was earlier in girls than boys, and girls progressed through puberty more quickly than boys (Figure 1). Additionally, girls self-reported more PLEs (total no. of PLEs reported and distress) at year 3 than boys. Correlations between youth-reported pubertal development and PLEs are outlined in Table 2.

**Table 1:** Descriptive Statistics for All Study Variables

	Full Sample, *N* = 11,718	Boys, *n* = 6134	Girls, *n* = 5624	χ^2^ or *t*	*p*	ES
Age, Baseline, Months	119.00 (7.49)	118.84 (7.51)	119.15 (7.47)	−2.26	.02	−0.04
Age, Year-3 Follow-Up, Months	154.98 (7.76)	155.19 (7.75)	154.74 (7.77)	−2.96	<.01	−0.06
Race, White	52.3%	53.2%	51.1%	7.71	.10	–

Covariates						
Caregiver Education, High School or Above	93.6%	93.7%	92.7%	4.86	.03	–
Premature Birth, ≥3 Weeks Early	13.5%	13.6%	13.4%	0.09	.76	–
Family History of Psychosis, ≥1 Family Member	10.3%	11.1%	10.4%	0.03	.85	–
Hormonal Contraceptive Use	–	–	3.1%	–	–	–
Total Psychotic-Like Experiences, Baseline	2.62 (3.55)	2.74 (3.64)	2.48 (3.45)	4.06	<.001	0.7
Distressing Psychotic-Like Experiences, Baseline	6.28 (10.57)	6.43 (10.66)	6.13 (10.47)	1.52	.13	0.03

Variables of Interest						
Pubertal Timing, Age, Years	12.80 (1.55)	13.80 (1.27)	11.70 (0.97)	101.24	<.001	1.85
Pubertal Tempo, Stages per Year	0.45 (0.15)	0.34 (0.09)	0.57 (0.11)	−127.11	<.001	−2.37
Adrenarchal Timing	12.54 (1.19)	13.22 (0.92)	11.80 (0.99)	80.61	<.001	1.49
Adrenarchal Tempo	0.45 (0.12)	0.40 (0.08)	0.51 (0.14)	−52.01	<.001	−0.98
Gonadarchal Timing	13.14 (1.74)	14.40 (1.27)	11.77 (0.96)	126.86	<.001	2.32
Gonadarchal Tempo	0.62 (0.24)	0.43 (0.11)	0.83 (0.14)	−167.36	<.001	−3.11
Total Psychotic-Like Experiences, Year 3	1.28 (2.48)	1.12 (2.25)	1.46 (2.70)	−6.88	<.001	−0.13
Distressing Psychotic-Like Experiences, Year 3	2.90 (6.62)	2.29 (5.44)	3.57 (7.67)	−9.65	<.001	−0.19

Pubertal Development						
	Baseline	Year 1	Year 2	Year 3		
Overall PDS						
Boys	1.65 (0.50)	1.70 (0.50)	1.88 (0.55)	2.19 (0.60)		
Girls	1.69 (0.54)	1.97 (0.62)	2.42 (0.67)	2.83 (0.63)		
Adrenarchal PDS						
Boys	1.64 (0.70)	1.70 (0.66)	1.96 (0.70)	2.31 (0.71)		
Girls	1.72 (0.72)	2.00 (0.74)	2.41 (0.71)	2.77 (0.64)		
Gonadarchal PDS						
Boys	1.42 (0.54)	1.46 (0.55)	1.61 (0.61)	1.93 (0.71)		
Girls	1.43 (0.57)	1.78 (0.77)	2.35 (0.95)	2.90 (0.81)		

For ease of interpretation, race/ethnicity, caregiver education, prematurity, and family history are dichotomized. For all analyses, race/ethnicity is coded with 5 levels: Asian, Black, Hispanic, Other, White; caregiver education is coded with 21 levels ranging from no education to doctoral degree; premature birth is coded as 1–13+ weeks, and family history of psychosis ranges from 0 to 6 family members.

ES, Cohen’s *d* effect size; PDS, Pubertal Development Scale.

**Table 2:** Correlations Among Study Variables

	Pubertal Timing	Pubertal Tempo	Adrenarchal Timing	Adrenarchal Tempo	Gonadarchal Timing	Gonadarchal Tempo	PLE Sum	PLE Distress
1. Pubertal Timing	–	0.04∗∗	0.84∗∗∗	0.40∗∗∗	0.87∗∗∗	−0.25∗∗∗	−0.17∗∗∗	−0.18∗∗∗
2. Pubertal Tempo	−0.26∗∗∗^a^	–	0.10∗∗∗	0.71∗∗∗	−0.04∗∗	0.68∗∗∗	−0.06∗∗∗	−0.06∗∗∗
3. Adrenarchal Timing	0.84∗∗∗^a^	0.00^a^	–	0.50∗∗∗	0.58∗∗∗	−0.15∗∗∗	−0.15∗∗∗	−0.16∗∗∗
4. Adrenarchal Tempo	0.19∗∗∗^a^	0.71∗∗∗^a^	0.45∗∗∗^a^	–	0.25∗∗∗	0.20∗∗∗	−0.11∗∗∗	−0.11∗∗∗
5. Gonadarchal Timing	0.86∗∗∗^a^	−0.33∗∗∗^a^	0.57∗∗∗^a^	0.09∗∗∗^a^	–	−0.34∗∗∗	−0.17∗∗∗	−0.17∗∗∗
6. Gonadarchal Tempo	−0.10∗∗∗^a^	0.75∗∗∗^a^	0.05∗∗∗^a^	0.39∗∗∗^a^	−0.26∗∗∗^a^	–	0.03∗	0.03∗
7. PLE Sum	−0.11∗∗∗^a^	−0.12∗∗∗^a^	−0.12∗∗∗^a^	−0.14∗∗∗^a^	−0.08∗∗∗^a^	−0.13∗∗∗^a^	–	0.99∗∗∗
8. PLE Distress	−0.11∗∗∗^a^	−0.12∗∗∗^a^	−0.12∗∗∗^a^	−0.14∗∗∗^a^	−0.08∗∗∗^a^	−0.13∗∗∗^a^	0.99∗∗∗^a^	–

∗*p* < .05, ∗∗*p* < .01, ∗∗∗*p* < .001.

PLE, psychotic-like experiences.

a Correlations for boys (bottom left). Correlations for girls are the values without the footnote (top right).

### Overall Pubertal Development and PLEs

**Figure 2:**
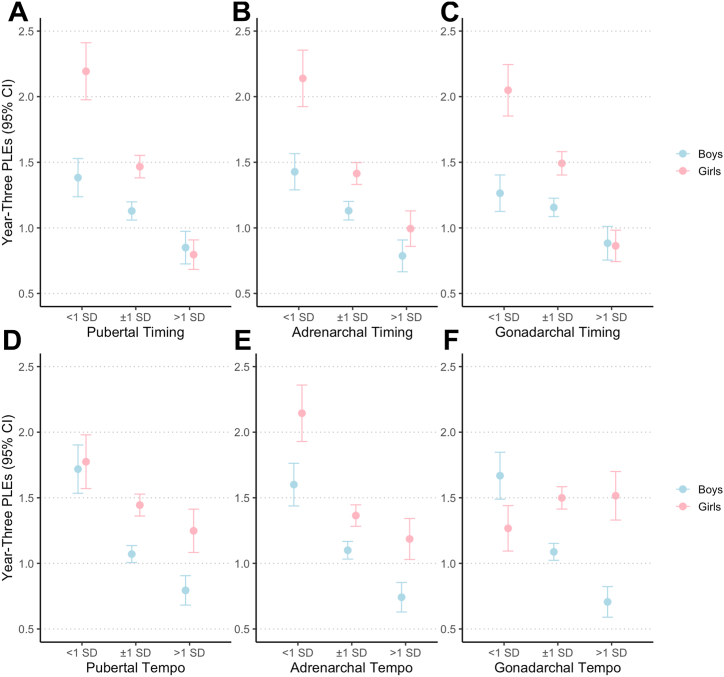
Categorical pubertal timing and tempo on year-3 psychotic-like experience (PLE) frequency. Results indicate a general pattern whereby earlier pubertal timing and faster pubertal tempo (across hormonal axes) confer risk for elevated PLEs relative to on-time/on-track developers, and later pubertal timing and slower pubertal tempo (across axes) confer potential resilience to experiencing PLEs at ∼13 years-old. Overall pubertal timing **(A)**, adrenarchal timing **(B)**, gonadarchal timing **(C)**, overall pubertal tempo **(D)**, adrenarchal tempo **(E)**, and gonadarchal tempo **(F)**. Note, for timing and tempo, respectively, <1 SD = “early timing” and “slower pace,” ±1 SD = “on-time” and “on-track pace,” >1 SD = “late timing” and “faster pace.” Estimates of year-3 pubertal development groups reflect group means before adjustment for covariates. See Figure S10 for plots with year-3 distressing PLEs, which demonstrate similar patterns to year-3 PLE sum scores.

Compared with girls with normative pubertal timing ($\bar{x}$_on-time_ PLEs = 1.47), girls with earlier pubertal timing reported more PLEs ($\bar{x}$_early_ PLEs = 2.19, β = 0.31, 95% CI [0.16 to 0.45]), whereas girls with later pubertal timing reported fewer PLEs ($\bar{x}$_delayed_ = 0.80, β = −0.52, 95% CI [−0.68 to −0.37]) (Figure 2A). A similar pattern of results emerged for PLE distress ($\bar{x}$_on-time_ = 3.53; $\bar{x}$_early_ = 5.57, β = 0.42, 95% CI [0.23 to 0.60]; $\bar{x}$_delayed_ = 1.92, β = −0.50, 95% CI [−0.69 to −0.32]) (Figure S1A). In girls, there was no effect of categorical pubertal tempo on the number of PLEs reported ($\bar{x}$_on-track_ = 1.44; $\bar{x}$_faster_ = 1.77, β = 0.05, 95% CI [−0.09 to 0.20]; $\bar{x}$_slower_ = 1.25, β = −0.06, 95% CI [−0.10 to 0.10]) or distress ($\bar{x}$_on-track_ = 3.50; $\bar{x}$_faster_ = 4.25, β = 0.07, 95% CI [−0.11 to 0.25]; $\bar{x}$_slower_ = −0.03, β = −0.50, 95% CI [−0.21 to 0.15]) (Figure 2D; Figure S1D).

Compared with boys with normative pubertal timing ($\bar{x}$_on-time_ PLEs = 1.13), boys with earlier pubertal timing reported more PLEs ($\bar{x}$_early_ = 1.38, β = 0.30, 95% CI [0.16 to 0.44]), whereas boys with later pubertal timing reported fewer PLEs ($\bar{x}$_delayed_ = 0.85, β = −0.25, 95% CI [−0.40 to −0.09]) (Figure 2A). Similar results were observed for PLE distress ($\bar{x}$_on-time_ = 2.32; $\bar{x}$_early_ = 2.72, β = 0.34, 95% CI [0.16 to 0.52]; $\bar{x}$_late_ = 1.75, β = −0.32, 95% CI [−0.52 to −0.13]) (Figure S1A).

In boys, compared with on-track developers ($\bar{x}$_on-track_ PLEs = 1.07, $\bar{x}$_on-track_ PLE distress = 2.15), slower pubertal tempo was associated with elevated PLE endorsement ($\bar{x}$_slower_ = 1.72, β = 0.40, 95% CI [0.25 to 0.55]) and higher PLE distress ($\bar{x}$_faster_ = 3.7, β = 0.50, 95% CI [0.31 to 0.69]). Faster pubertal tempo was associated with reduced PLE endorsement ($\bar{x}$_faster_ = 0.79, β = −0.23, 95% CI [−0.39 to −0.07]) and lower PLE distress ($\bar{x}$_slower_ = 1.51, β = −0.30, 95% CI [−0.49 to 0.10]) (Figure 2D; Figure S1D).

In girls and boys, continuously measured pubertal timing, pubertal tempo, and their interaction were not associated with the number of PLEs reported or distress scores (Table S1).

### Adrenarchal Development and PLEs

Compared with girls with normative adrenarchal timing ($\bar{x}$_on-time_ PLEs = 1.41), girls with earlier adrenarchal timing reported more PLEs ($\bar{x}$_early_ = 2.14, β = 0.26, 95% CI [0.10 to 0.42]), whereas girls with later adrenarchal timing reported fewer PLEs ($\bar{x}$_early_ = 1.00, β = −0.32, 95% CI [−0.47 to −0.17]) (Figure 2B). A similar pattern of results emerged for PLE distress ($\bar{x}$ = 3.42; $\bar{x}$_early_ = 5.48, β = 0.35, 95% CI [0.14 to 0.55]; $\bar{x}$_delayed_ = 2.29, β = −0.37, 95% CI [−0.55 to −0.19]). In girls, there was no effect of categorical adrenarchal tempo on number of PLEs reported ($\bar{x}$_on-track_ = 1.36; $\bar{x}$_faster_ = 2.14, β = 0.07, 95% CI [−0.08 to 0.22]; $\bar{x}$_slower_ = 1.19, β = 0.06, 95% CI [−0.10 to 0.21]) or distress ($\bar{x}$_on-track_ = 3.28; $\bar{x}$_faster_ = 5.42, β = 0.12, 95% CI [−0.07 to 0.31]; $\bar{x}$_slower_ = 2.93, β = 0.12, 95% CI [−0.07 to 0.30]) (Figure 2E; Figure S1E).

**Figure 3:**
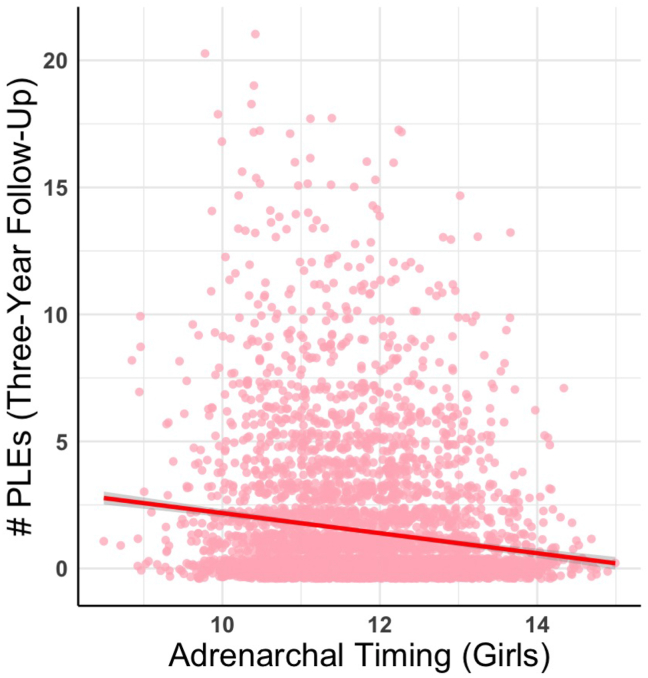
Adrenarchal timing in girls and number of psychotic-like experiences (PLEs). Later adrenarchal timing was associated with reduced PLEs at 3-year follow-up.

In girls, continuously measured adrenarchal timing was negatively associated with number of PLEs (β = −0.21, 95% CI [−0.40 to −0.02]) (Figure 3) and distress (β = −0.28, 95% CI [−0.52 to −0.05]). Neither adrenarchal tempo nor the interaction between adrenarchal timing and tempo were associated with number of PLEs reported or distress (Table S2).

Compared to boys with normative adrenarchal timing ($\bar{x}$_on-time_ PLEs = 1.13), earlier adrenarchal timing was not associated with different PLE endorsement. Later adrenarchal timing was associated with reporting fewer PLEs ($\bar{x}$_delayed_ = 0.79, β = −0.22, 95% CI [−0.37 to −0.07]) (Figure 2B). Similar results were observed for PLE distress ($\bar{x}$_on-time_ = 2.32; $\bar{x}$_early_ = 2.86, β = 0.12, 95% CI [−0.07 to 0.31]; $\bar{x}$_delayed_ = 1.60, β = −0.27, 95% CI [−0.46 to −0.08]) (Figure S1B). In boys, slower adrenarchal tempo was not associated with the number of PLEs reported or PLE distress compared with on-track developers ($\bar{x}$_on−track_ PLE reporting = 1.10, $\bar{x}$_on-track_ PLE distress = 2.25). In boys, faster adrenarchal tempo was associated with reporting fewer PLEs ($\bar{x}$_faster_ = 0.74, β = −0.21, 95% CI [−0.37 to −0.06]) and less PLE distress ($\bar{x}$_faster_ = 1.46, β = −0.25, 95% CI [−0.44 to −0.06]) (Figure 2E; Figure S1E).

**Figure 4:**
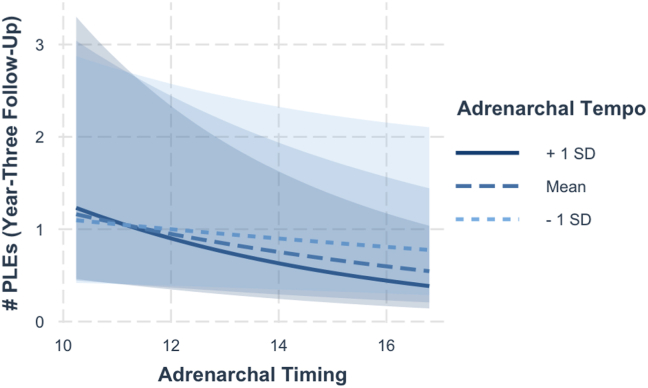
Interaction between adrenarchal timing and adrenarchal tempo in boys. Year-3 psychotic-like experiences (PLEs) were greater in boy youth with later adrenarchal timing and slower adrenarchal tempo, with no main effects observed for either (Table S2). Interaction analysis was conducted with continuous metrics of adrenarchal timing and tempo. For display purposes only, adrenarchal tempo was categorized into ±1 SD. Analyses with distressing PLEs did not show the same interaction (*p* > .05).

In boys, neither continuous adrenarchal timing nor adrenarchal tempo were associated with the number of PLEs reported (Table S2). The interaction between adrenarchal timing and tempo was significant in boys (β = −0.80, 95% CI [−1.50 to −0.10]), suggesting that boys with delayed adrenarchal timing and slower adrenarchal tempo reported more PLEs (Figure 4). No significant results were observed for distressing PLEs (Table S2).

### Gonadarchal Development and PLEs

Compared with girls with normative gonadarchal timing ($\bar{x}$_on-time_ = 1.49), girls with earlier gonadarchal timing reported more PLEs ($\bar{x}$_early_ = 2.05, β = 0.29, 95% CI [0.14 to 0.43]), whereas girls with later gonadarchal timing reported fewer PLEs ($\bar{x}$_delayed_ = 0.86, β = −0.47, 95% CI [−0.64 to −0.30]) (Figure 2C). A similar pattern of results emerged for PLE distress ($\bar{x}$_on-time_ = 3.60; $\bar{x}$_early_ = 5.15, β = 0.36, 95% CI [0.17 to 0.54]; $\bar{x}$_delayed_ = 2.10, β = −0.48, 95% CI [−0.69 to −0.28]) (Figure S1C). In girls, there was no effect of categorical gonadarchal tempo on the number of PLEs reported ($\bar{x}$_on-track_ = 1.50; $\bar{x}$_faster_ = 1.27, β = 0.02, 95% CI [−0.13 to 0.18]; $\bar{x}$_slower_ = 1.52, β = 0.06, 95% CI [−0.08 to 0.21]) or distress ($\bar{x}$_on-track_ = 3.68; $\bar{x}$_faster_ = 3.07, β = 0.03, 95% CI [−0.16 to 0.21]; $\bar{x}$_slower_ = 3.65, β = 0.03, 95% CI [−0.16 to 0.21]) (Figure 2F; Figure S1F).

Compared with boys with normative gonadarchal timing ($\bar{x}$_on-time_ = 1.16), boys with earlier gonadarchal timing reported more PLEs ($\bar{x}$_early_ = 1.26, β = 0.23, 95% CI [0.09 to 0.37]). In boys, later gonadarchal timing was associated with reporting fewer PLEs ($\bar{x}$_delayed_ = 0.88, β = −0.29, 95% CI [−0.43 to −0.14]) (Figure 2C). Similar results were observed for PLE distress ($\bar{x}$_on-time_ = 2.36; $\bar{x}$_early_ = 2.45, β = 0.25, 95% CI [0.08 to 0.43]; $\bar{x}$_delayed_ = 1.91, β = −0.33, 95% CI [−0.51 to −0.15]) (Figure S1C). Boys with slower gonadarchal tempo reported more PLEs ($\bar{x}$_slower_ = 1.67, β = 0.32, 95% CI [0.17 to 0.47]) and distress ($\bar{x}$_slower_ = −3.56, β = 0.41, 95% CI [0.22 to 0.60]) compared with on-track developers ($\bar{x}$_on-track_ PLEs = 1.09, $\bar{x}$_on-track_ PLE distress = 2.19). Faster gonadarchal tempo was associated with reporting fewer PLEs ($\bar{x}$_faster_ = 0.71, β = −0.50, 95% CI [−0.68 to −0.32]) and less PLE distress ($\bar{x}$_faster_ = 1.41, β = −0.53, 95% CI [−0.75 to −0.31]) (Figure 2F; Figure S1F).

In girls and boys, continuously measured gonadarchal timing, gonadarchal tempo, and the interaction were not associated with the number of PLEs reported or distress (Table S3).

### Sex Differences in the Relationship Between Pubertal Indicators and PLEs

To assess potential sex differences in the relationship between indicators of pubertal development and PLEs, boys and girls were run in the same negative binomial mixed-effects model. Generally, these models showed a significant interaction wherein earlier pubertal, adrenarchal, and gonadarchal timing in girls and slower pubertal, adrenarchal, and gonadarchal tempo in boys impacted PLEs to a greater extent. Upon closer inspection, these interactions were likely driven by normative and pronounced sex differences in pubertal timing and tempo.

## Discussion

**Figure 5:**
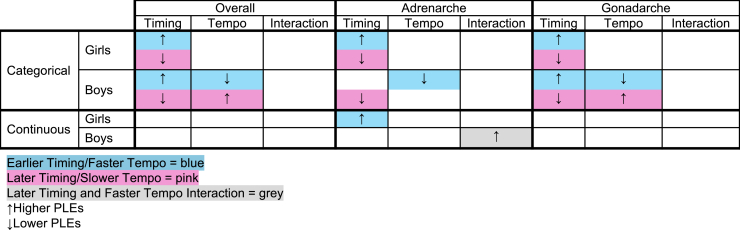
Summary of significant findings. PLE, psychotic-like experience.

Puberty matters for psychosis (2,14,19,22,25,51, 52, 53). However, past work has been limited by the conceptualization and assessment of puberty as a single event (i.e., age of menarche) rather than a biopsychosocial process that unfolds over years. We used advanced longitudinal approaches in a large nationally representative dataset to determine how pubertal timing, tempo, and their interactions were related to PLEs separately in boys and girls. Figure 5 summarizes the major findings. For the first time, we demonstrated that earlier pubertal timing was associated with increased reporting of PLEs 3 years later in boys and girls. We evaluated the associations of pubertal timing and tempo with PLEs using a categorical measure to capture onset relative to same-age peers and a continuous measure of timing to capture absolute age of onset. This multimethod approach allowed us to 1) isolate the contribution of specific hormonal axes and 2) determine whether risk for PLEs was associated with socially meaningful differences in timing (i.e., being “early” or “late”) or biological differences in timing (i.e., absolute age).

Specifically, when considered as a categorical variable (reference group = “on-time”), earlier development was associated with many PLEs, and later development was associated with few PLEs. Findings were consistent across hormonal axes and sex. In boys only, we demonstrated a specific effect of tempo. Faster tempo was associated with fewer PLEs, and slower tempo was associated with more PLEs; findings were generally consistent across hormonal axes. When considered as a continuous variable, earlier timing was only associated with more PLEs within the adrenal axis. Although there were significant sex differences, our analysis suggested that the differences were attributable to sex differences in normative pubertal timing and tempo rather than true moderation by sex. These findings are contextualized below within prevailing theories of pubertal development.

Consistent with the maturational disparity hypothesis, we observed that “early” pubertal timing compared with same-age peers was associated with reporting more PLEs. Associations were more robust for categorical timing than absolute age of onset, further supporting the maturational deviance hypothesis, which centers social deviance from peers rather than age (54). This finding is contrary to work suggesting that early puberty is protective for women (i.e., associated with later psychosis onset) (23,24,55); however, such findings have been based on the timing of menarche alone rather than the complex biopsychosocial sequelae of pubertal events as was done here. Indeed, emerging research suggests that associations between pubertal timing and well-being generally are mediated by psychosocial influences such as peer influence (56), body image concerns (57), and emotion regulation styles (58). Future research should examine these and other psychosocial and cognitive mechanisms longitudinally to clarify how early pubertal timing increases risk for PLEs specifically and identify potential targets for early intervention.

Although the results primarily support the maturational disparity hypothesis, the absence of off-time effects and the protective effect of later development on PLEs suggest that earlier hormone exposure may directly increase risk for later PLEs as well. This finding is consistent with prior research that suggests that youth who develop early have prolonged exposure to sex hormones over the lifespan, which can be protective for some outcomes (24,59) but detrimental for others (13,60). Additionally, we found that slower pubertal tempo (i.e., slower increases in sex hormone levels) was associated with more PLEs; this finding was only evident in boys but suggests that future research should assess initial exposure to sex hormones and changes that occur over the course of puberty. Indeed, there is accumulating evidence that youth who start puberty earlier than their peers have distinct profiles of hormone exposure (29,61,62), suggesting that differences between the hormonal profiles of earlier and later developers may not just dissipate over time (63,64). In particular, extended exposure to cortisol, which can both regulate and be regulated by sex hormones, has been implicated in the earlier onset of psychosis and psychotic phenotypes, such as hippocampal development (19).

The findings from the gonadal axis and adrenal axis were mostly consistent with those derived using the overall PDS measure with one notable exception: Earlier adrenarche was associated with number of PLEs among girls when considering absolute age of onset. This finding suggests that adrenal hormones such as DHEA may play a distinct role in shaping risk for psychopathology during adolescence (65,66). This finding contrasts with existing research, which has mostly focused on gonadal hormones such as estrogen, which is hypothesized to play a key neuroprotective role, especially for positive symptoms (14,19), and testosterone, which has been linked to increased negative symptoms (2,14,52,53). To date, adrenal hormones have received little attention in relation to PLEs despite their relevance to stress responses (67,68) and emotion regulation (69,70). While we urge caution in interpreting this single finding, it highlights the need for further investigation of sex hormones beyond estrogen and testosterone (29).

Importantly, results were similar regardless of whether PLE sum scores or PLE distress scores were used. Associations between pubertal timing and tempo and PLE sum scores suggests that early pubertal timing and slower pubertal tempo may confer risk for PLEs through very basic processes (e.g., perceptual and cognitive system development), whereby aberrations map onto the experience of PLEs. The impact of pubertal metrics on PLE sum scores may be mediated by the organizational effects of pubertal steroid hormones, with receptor sites densely populated in key brain regions with protracted development (e.g., prefrontal cortex, hippocampus) (71,72). Additionally, associations between pubertal timing and tempo and distressing PLEs suggests that these developmental processes may confer risk for PLEs through higher-level processes (e.g., stress, peer relationships) as well. For example, earlier-developing youth may experience discordance between their physical development (e.g., breast budding, hair growth) and their cognitive development, prompting a change in the perception of their and others’ view of themselves. Taken together, these findings suggest that future work is warranted to determine the roles of hormonal, neural, social, emotional, and self-perception factors.

The current work is a foundational step in clarifying the role of puberty in the emergence of psychosis in boys and girls. This study included the most time points used in a study on puberty and PLEs to date but was limited by the early age of participants in the ABCD Study. Although there was sufficient variability in timing and tempo, future analyses could investigate the long-term impact of pubertal development on PLEs into adulthood. Another advantage of the current study is the use of continuous and categorical measures of pubertal timing. The association between absolute age of onset and PLEs reflects the linear relationship between timing and risk for PLEs, which may not capture meaningful developmental differences between youth (73). In contrast, the categorical measure groups individuals into socially meaningful categories (i.e., being early or late relative to peers), which increases its power and sensitivity to detect effects (28). This finding highlights the value of considering both biological and social dimensions of timing.

Regarding limitations, we did not directly investigate changes in brain structure or function in the current study or changes in hormones. While the direct investigation of mechanisms was beyond the scope of this study, our findings clarify the relative contribution of different hormonal axes within prevailing pubertal theories, thereby providing a foundation for future mechanistic work. Such studies may examine brain and hormonal changes as independent outcomes or test them as mediators or moderators in longitudinal models to better understand the organizational and activational roles of puberty in shaping PLEs and related functional outcomes. Similarly, the current analyses only used PLEs from the year-3 follow-up. Despite controlling for baseline PLEs in the current analysis, future work should examine the effects of pubertal timing on longitudinal trajectories of PLEs (e.g., latent growth analysis, growth mixture modeling). Finally, the PQ-BC, while multifaceted, is solely representative of the positive spectra of symptomology observed in emerging psychotic disorders. More comprehensive measures of prodromal syndromes may provide additional clarity on the role of puberty in positive symptomology versus negative symptoms (1).

### Conclusions

Our findings suggest a central role of puberty in longstanding organizational processes on psychopathology. To our knowledge, in this first-ever longitudinal analysis of pubertal development and PLEs, we demonstrated that early pubertal timing and faster tempo (boys only) were associated with elevated PLEs 3 years later. This work is critical given significant limitations of prior work, global rates of earlier pubertal development (74,75), and increased rates of PLEs and psychosis. Understanding the contributions of distinct metrics of pubertal development and the unique experiences associated with a youth’s pubertal onset can help clarify the roles of hormonal, neural, and social factors in psychopathology onset, identification, and prevention efforts for children and adolescents at greatest risk.

## Supplementary Material

Figure S1 and Tables S1–S3

Key Resources Table
