# Supplementary material for: Early Pubertal Development Is a Risk Factor for Psychotic-Like Experiences in Boys and Girls
Source: Biol Psychiatry Glob Open Sci. 2025 Oct 31;6(2):100647. doi: 10.1016/j.bpsgos.2025.100647 (PMC12799914; doi:10.1016/j.bpsgos.2025.100647)
Supplement: Figure S1 and Tables S1–S3 [file mmc1.pdf]

SUPPLEMENTARY INFORMATION

Early Pubertal Development Is a Risk Factor for Psychotic-Like Experiences in Boys and Girls

Larson *et al.*

Supplemental Figure 1: Categorical Pubertal Timing and Tempo on Year-Three Psychotic-Like Experience Distress .... p. 2

Supplemental Table 1: Main and interaction effects of continuously measured overall pubertal timing and tempo on year-three PLE sum and distress scores.............................. p. 3

Supplemental Table 2: Main and interaction effects of continuously measured adrenarchal timing and tempo on year-three PLE sum and distress scores.............................. p. 4

Supplemental Table 3: Main and interaction effects of continuously measured gonadarchal timing and tempo on year-three PLE sum and distress scores.............................. p. 5

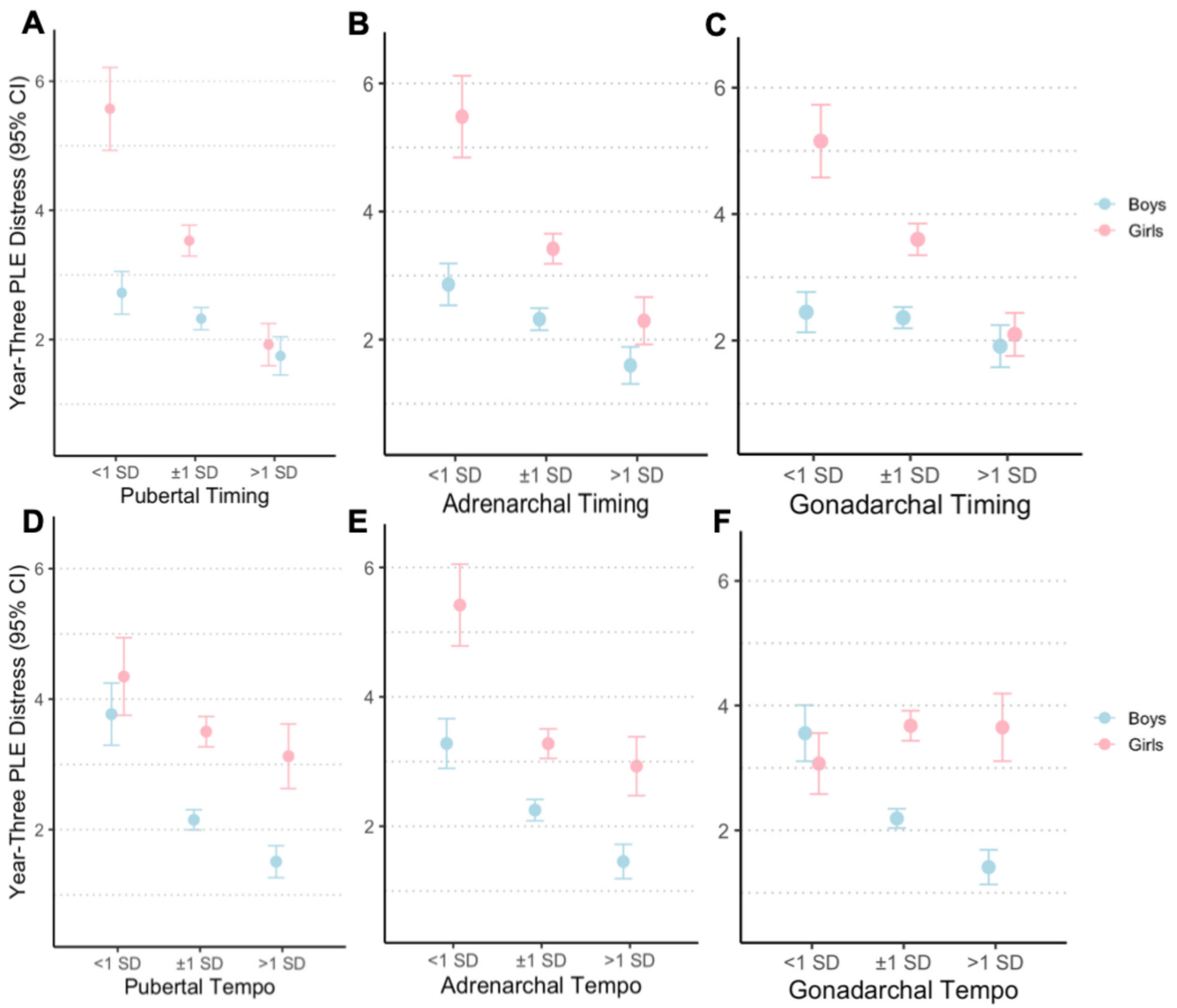

Supplemental Figure 1: Categorical Pubertal Timing and Tempo on Year-Three Psychotic-Like Experience Distress. Results indicate a general pattern whereby earlier pubertal timing and faster pubertal tempo (across hormonal axes) confer risk for elevated distressing PLEs relative to on-time/on-track developers, and later pubertal timing and slower pubertal tempo (across axes) confer potential resilience to experiencing distressing PLEs at ~13 years-old. Overall Pubertal Timing (**A**), Adrenarchal Timing (**B**), Gonadarchal Timing (**C**), Overall Pubertal Tempo (**D**), Adrenarchal Tempo (**E**), and Gonadarchal Tempo (**F**). Note, for timing and tempo respectively, <1SD="early timing" and "slower pace", ±1SD="on-time" and "on-track pace", >1SD="late timing" and "faster pace'). Estimates of year-three pubertal development groups reflect group means before adjustment for covariates. PLEs=psychotic-like experiences. SD=standard deviation. See Figure 1 for plots with year-three PLE sum scores, which demonstrate similar patterns to year-three PLE sum scores.

Supplemental Table 1. Main and interaction effects of continuously measured overall pubertal timing and tempo on year-three PLE sum and distress scores.				
Sex	Pubertal Indicator	PLE Indicator	Beta	95% CI
Girls	Pubertal Timing	PLE Sum	-0.21	-0.47, 0.05
Girls	Pubertal Timing	PLE Distress	-0.25	-0.58, 0.07
Girls	Pubertal Tempo	PLE Sum	1.48	-4.17, 7.13
Girls	Pubertal Tempo	PLE Distress	1.23	-5.77, 8.24
Girls	Timing*Tempo	PLE Sum	-0.14	-0.63, 0.34
Girls	Timing*Tempo	PLE Distress	-0.12	-0.72, 0.47
Boys	Pubertal Timing	PLE Sum	-0.04	-0.19, 0.11
Boys	Pubertal Timing	PLE Distress	-0.06	-0.25, 0.13
Boys	Pubertal Tempo	PLE Sum	2.46	-3.46, 8.39
Boys	Pubertal Tempo	PLE Distress	2.32	-5.31, 9.97
Boys	Timing*Tempo	PLE Sum	-0.33	-0.76, 0.10
Boys	Timing*Tempo	PLE Distress	-0.36	-0.92, 0.19
Note: Association is considered significant if the 95% CI does not include 0.				

Supplemental Table 2. Main and interaction effects of continuously measured adrenarchal timing and tempo on year-three PLE sum and distress scores.				
Sex	Pubertal Indicator	PLE Indicator	Beta	95% CI
Girls	Adrenarchal Timing	PLE Sum	-0.21	-0.40, -0.02
Girls	Adrenarchal Timing	PLE Distress	-0.28	-0.52, -0.05
Girls	Adrenarchal Tempo	PLE Sum	0.87	-3.45, 5.20
Girls	Adrenarchal Tempo	PLE Distress	0.34	-5.13, 5.81
Girls	Timing*Tempo	PLE Sum	-0.08	-0.45, 0.29
Girls	Timing*Tempo	PLE Distress	-0.03	-0.50, 0.44
Boys	Adrenarchal Timing	PLE Sum	0.20	-0.07, 0.47
Boys	Adrenarchal Timing	PLE Distress	0.19	-0.15, 0.53
Boys	Adrenarchal Tempo	PLE Sum	8.88	-0.04, 17.80
Boys	Adrenarchal Tempo	PLE Distress	9.30	-1.83, 20.44
Boys	Timing*Tempo	PLE Sum	-0.84	-1.48, -0.11
Boys	Timing*Tempo	PLE Distress	-0.80	-1.70, 0.02
Note: Association is considered significant if the 95% CI does not include 0.				

Supplemental Table 3. Main and interaction effects of continuously measured gonadarchal timing and tempo on year-three PLE sum and distress scores.				
Sex	Pubertal Indicator	PLE Indicator	Beta	95% CI
Girls	Gonadarchal Timing	PLE Sum	-0.08	-0.35, 0.19
Girls	Gonadarchal Timing	PLE Distress	-0.17	-0.49, 0.15
Girls	Gonadarchal Tempo	PLE Sum	2.84	-1.36, 7.04
Girls	Gonadarchal Tempo	PLE Distress	1.82	-3.18, 6.83
Girls	Timing*Tempo	PLE Sum	-0.25	-0.61, 0.10
Girls	Timing*Tempo	PLE Distress	-0.17	-0.60, 0.25
Boys	Gonadarchal Timing	PLE Sum	-0.061	-0.20, 0.07
Boys	Gonadarchal Timing	PLE Distress	-0.03	-0.22, 0.15
Boys	Gonadarchal Tempo	PLE Sum	0.35	-4.22, 4.91
Boys	Gonadarchal Tempo	PLE Distress	1.44	-4.62, 7.50
Boys	Timing*Tempo	PLE Sum	-0.15	-0.48, 0.17
Boys	Timing*Tempo	PLE Distress	-0.26	-0.68, 0.16
Note: Association is considered significant if the 95% CI does not include 0.				
